# Robotic-assisted partial nephrectomy with sequential clamping of segmental renal arteries for multiple ipsilateral renal tumors: initial outcomes

**DOI:** 10.1186/s12894-019-0451-y

**Published:** 2019-05-03

**Authors:** Jie Yang, Jia-dong Xia, Jian-xin Xue, Ning-hong Song, Chao Liang, Di Xi, Ya-min Wang, Zeng-jun Wang

**Affiliations:** Department of Urology, First Affiliated Hospital of Nanjing Medical University, Nanjing, 210029 Jiangsu China

**Keywords:** Multiple ipsilateral renal tumors, Robotic-assisted partial nephrectomy, Segmental renal artery, Dual-source computed tomography, Sequential clamping, Estimated glomerular filtration rate

## Abstract

**Background:**

To assess the technical feasibility and outcomes of robotic-assisted partial nephrectomy (RPN) with sequential segmental renal artery (SRA) clamping for multiple ipsilateral renal tumors (MIRTs).

**Methods:**

From April 2016 to February 2018, consecutive eleven cases successfully underwent RPN with sequential SRA clamping under the guidance of dual-source computed tomography (DSCT).

**Results:**

Ten cases had two lesions and two cases had three at the ipsilateral kidneys. The mean size and the mean R.E.N.A.L score for the dominant lesion of single case were 3.3 cm and 5.7, respectively. Twenty-two lesions (84.6%) had one target SRA and four (15.4%) had two target SRAs. Satisfactory ischemic areas were achieved by sequentially clamping two (81.8%) or three (18.2%) target SRAs with mean clamping time of 18.8 (15.0–27.0) min for single lesion, and the mean of total clamping time for single case was 37.5 (32.0–52.0) min. Only the complications of grade 1–2 were found and no positive surgical margin was discovered. The mean follow-up time was 5.4 months and no local recurrence or metastasis was found. The mean postoperative eGFR was 71.2 ml/minute/1.73m^2^ that was only an insignificant reduction (9.3%) compared with the preoperative baseline.

**Conclusion:**

This novel nephron-sparing technique, RPN with sequential SRA clamping, represents a good alternative for selected patients with MIRTs. With the guidance of DSCT and skilled robotic experience, this technique is feasible and can maximize renal function preservation. Large-scale multicenter clinical studies are still needed to further prove these initial outcomes.

**Electronic supplementary material:**

The online version of this article (10.1186/s12894-019-0451-y) contains supplementary material, which is available to authorized users.

## Background

Multiple ipsilateral renal tumors (MIRTs) are characterized as at least two tumor foci in the same kidney, separated by normal tissue [1, 2]. The exact incidence of MIRTs was currently unreported. According to the available related data, we conservatively estimated the incidence between 4.5 and 7.9% [3]. Radical nephrectomy (RN) has been regarded as the standard treatment in previous studies. Unfortunately, patients with MIRTs would be highly predisposed to form contralateral renal tumors at the morbidity of about 5% [4, 5], thus RN is definitely not the optimal treatment for MIRTs.

Since 2003, a series of reports have suggested that nephron-sparing surgery (NSS) can provide a reliable oncologic cure and better postoperative function outcomes compared with RN in selected MIRT patients [5–7]. Meanwhile, robot-assisted partial nephrectomy (RPN) was reported in 2004 along with the growing confidence in robotic-assisted laparoscopic technique [8, 9]. Then Rogers et al. firstly reported RPN with main renal artery (MRA) clamping for the treatment of MIRTs in 2008 [10]. However, the excision of multiple lesions inevitably requires more MRA clamping time, which will aggravate the ischemic/ reperfusion injury of normal spared nephron [11–13].

In order to further optimize the surgery treatment of MIRTs, we originally propose a novel clamping procedure of lesion-feeding arteries in RPN, the sequential clamping of precise segmental renal arteries (SRAs) [14]. Under the guidance of dual-source computed tomography (DSCT) [11–14], the technique has been successfully applied in the procedure of laparoscopic partial nephrectomy (LPN) for the treatment of MIRT in our center since 2010 and reported in 2017 [15]. But precise hilar microdissection required by sequential SRA clamping and one-time resection/renorrhaphy for multiple renal tumors are two major challenges of the technique in laparoscopic procedure. Robot-assisted platform can greatly help to overcome these challenges because of its two technical advantages. The first is its clearer 3D- field of vision with greater magnification than ordinary laparoscopic surgery, which can facilitate the identification of small branches of the renal arteries [10]. The other one is its more precise operating angle that can realize the quick resection and renorrhaphy of multiple lesions [9]. The aim of this study is to describe the application of sequential SRA clamping technique in the procedure of RPN and assess the technical feasibility for the treatment of MIRTs. Herein, we present our initial experience and short-term outcomes of this technique in a series of twelve patients.

### Methods

#### Inclusion criteria

From April 2016 to February 2018, consecutive twelve MIRT cases, preoperatively diagnosed as renal cell carcinoma (RCC) or renal angiomyolipomas (AMLs) by computed tomography (CT) (Additional file 1: Figure S1), underwent RPN with sequential SRA clamping in our center. Under the guidance of preoperative DSCT, all cases had at least two SRAs feeding different lesions (Additional file 1: Figure S1C). All cases had normal contralateral renal function, evaluated by Gate’s method before operation [16]. No case had bilateral or hilar lesions or metastatic foci, and no case had unacceptable anesthetic/operative risk based on the primary operator’s judgment.

#### Surgical methods

**Fig. 1:**
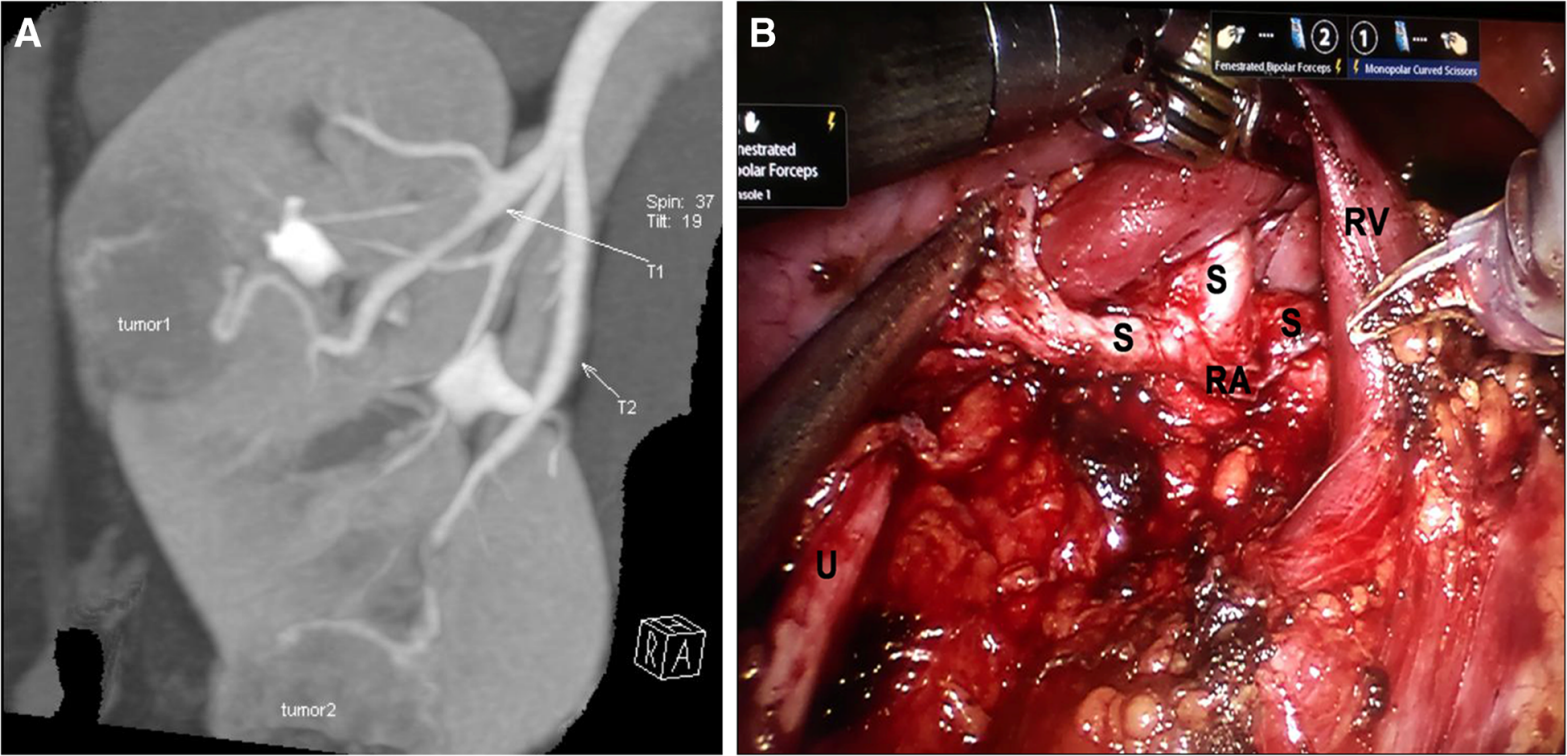
The preoperative picture (**a**) of dual-source computed tomography (DSCT) angiography precisely predicts the two segmental renal arteries (SRAs) (T1 and T2) feeding two different lesions (tumor1 and tumor2) in the right kidney respectively, as confirmed by the hilar anatomy during robot-assisted partial nephrectomy (RPN) (**b**). T = target SRA

All RPNs with sequential SRA clamping were performed by an experienced surgeon (Z.W.) independently through a transperitoneal approach [17, 18]. The standard SRA clamping technique was described in our previous studies on LPN [11–15]. Before operation, an additional DSCT was taken out to establish three-dimensional (3D) dynamic renal vascular models for every case (Fig. 1a). Thus, the anatomic relationship of SRAs with lesions was obtained and the feeding SRAs of every lesion could be precisely identified before operation (Fig. 1a).

**Fig. 2:**
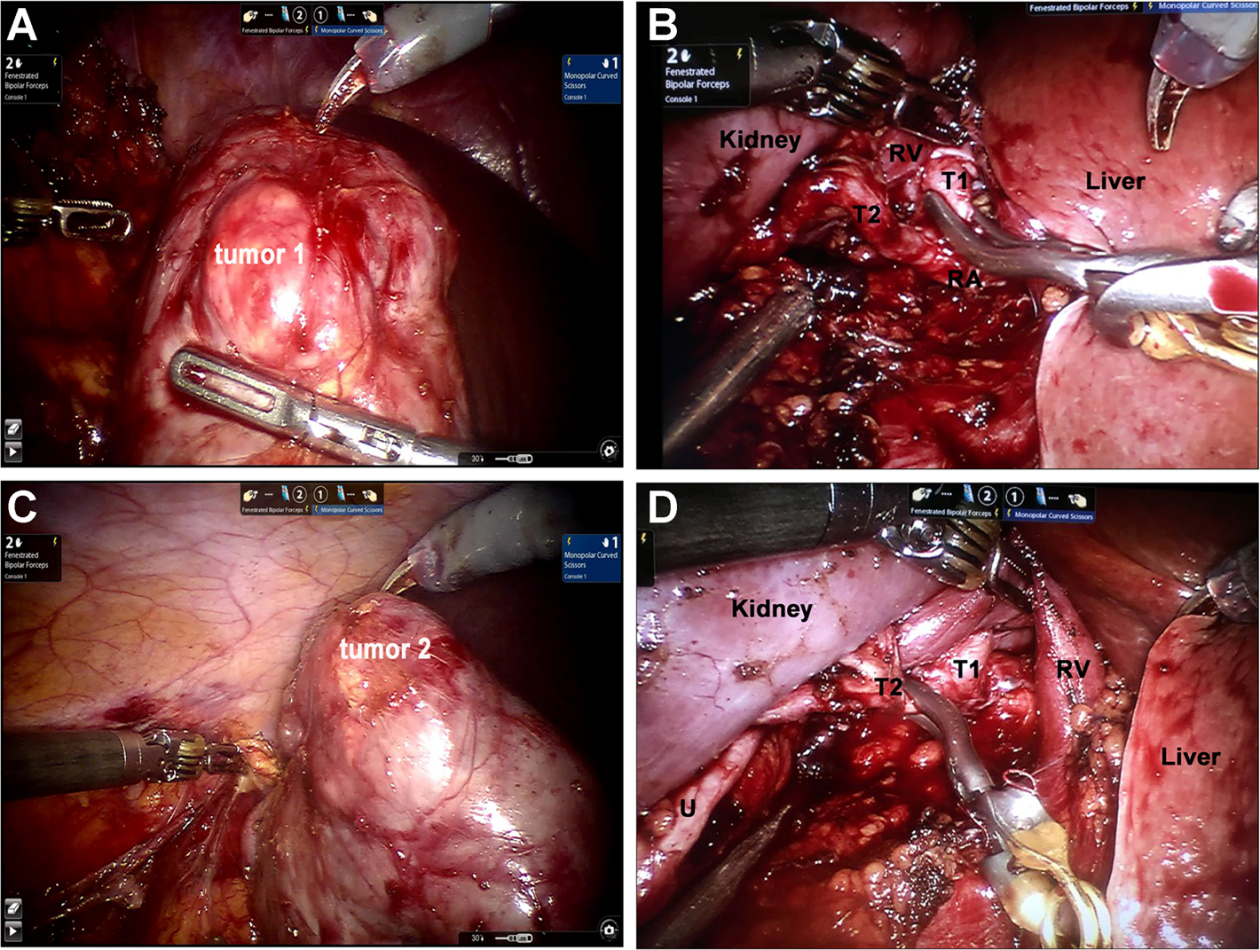
Sequentially clamping the target segmental renal artery (SRA) 1 (**b**) and 2 (**d**) respectively for the ipsilateral high-score (**a**) and low-score (**c**) tumors in the right kidney. T = target SRA; U = ureter; RA = renal artery; RV = renal vein


Patients under general anesthesia were placed in the modified flank position at 60 degrees (Additional file 2: Figure S2A). The placement of five ports in the abdomen region (Additional file 2: Figure S2B) and subsequently docking with the DaVinci robot arms (Intuitive Surgical, Sunnyvale, CA, USA) were same as previously reported by other centers [17, 18]. When the MRA was identified at the renal hilum, further dissection was performed to isolate target SRAs for precisely clamping with neurosurgical bulldogs [11–15] (Fig. 1b). Following an easy-to-difficult procedure to expose each tumor (Fig. 2a, c), we first handled the renal tumor with a lower R.E.N.A.L score. With precisely clamping of the feeding SRA, a satisfying local ischemic area around the low-score lesion was achieved, and then we excised the tumor closely around its capsule with a margin of 1–2 mm normal parenchyma (Fig. 2a, b). If the ischemic area could not encompass the whole lesion after single branch was clamped, multiple SRAs would be done. When the excision was completed, renorrhaphy was performed to achieve haemostasis and the closure of incised calices using 2–0/3–0 barbed sutures (QUILL SRS, PA, USA) and hem-o-lok clips (Sanlian Xinghai Medical Innovation Co. Ltd., Jiangsu, China) before the feeding SRA was unclamped [17, 18]. It was also necessary to clamp additional SRAs when there was arterial bleeding from lesion beds. If there was excessive bleeding in the surgical field of vision, or clamping multiple SRAs could not obtain satisfactory ischemic area, conversion to MRA clamping or open procedure was required. Then, another high-score lesion with its feeding SRA was dealt with following the above procedure (Fig. 2c, d) (Additional file 3: Video S1).

**Additional file 3**: Video 1. Robotic-Assisted partial nephrectomy with sequential clamping of segmental renal arteries for two ipsilateral renal tumors in the right kidney. (WMV 121213 kb)


#### Perioperative evaluations

We reviewed and recorded demographics, lesion characteristics, laboratory results, operative outcomes, and pathology reports. Serum creatinine (Scr) and estimated glomerular filtration rate (eGFR) at baseline and on the postoperative 30th day were applied to assess the function outcomes. All patients underwent a preoperative radiologic evaluation with contrast-enhanced CT to delineate the renal lesions and its size, as well as invasion depth. In addition, to delineate the renal vascular segmentation and indicate the target artery, CT angiography (CTA) with 3D reconstruction model was imaged before operation.

#### Data analysis

Complications were recorded according to the modified Clavien-Dindo classification [19]. eGFR was calculated using the Modification of Diet in Renal Disease equation: GFR in mL/min/1.73 m^2^ = 186 × Scr^− 1.154^ × age^− 0.203^× (0.742 if female) [20]. The highest one of R.E.N.A.L nephrometry scores was considered for each patient [21]. Descriptive continuous variables were expressed with mean ± standard deviation. Descriptive categorical variable were summarized using frequencies and percentages. All statistical calculations were performed with the SPSS Statistics software v.19 (IBM, Armonk, NC, USA).

## Results

### Patient and lesion characteristics

The demographic and preoperative characteristics of twelve patients are summarized in Additional file 4: Table S1. The mean age was 62.7 years, and mean preoperative Scr and eGFR were 92.8 (range: 74.1–122.3) μmol/l and 78.5 (range: 54.8–113.0) ml/minute/1.73m^2^, respectively. Ten cases had two lesions and two cases had three at the ipsilateral kidneys. Nine cases were diagnosed with RCCs of stage cT1a-cT1b and three were with AMLs by preoperative CT scanning. The mean size for single lesion was 2.7 cm, and the mean size and the mean R.E.N.A.L score for the dominant lesion of single case was 3.3 cm and 5.7, respectively. Two cases (16.7%) had a posterior lesion, six cases (50.0%) had at least a mesophytic lesion and one (8.3%) had an endophytic lesion. According to the 3D-reconstruction models of preoperative DSCT, twenty-two lesions (84.6%) had one target SRA and four (15.4%) had two target SRAs.

### Operative outcomes

RPNs were completed in all twelve cases without conversion to open procedure or total nephrectomy. Eleven of twelve cases were completed successfully with sequential SRA clamping, and one patient converted to MRA clamping after three target SRAs were clamped but without satisfactory bleeding control. The operative outcomes of the eleven cases are summarized in Table 1. The mean operative time was 91.7 (range: 72.0–155.0) min with mean estimated blood loss (EBL) of 150.0 (range: 50.0–350.0) ml, and no patient underwent any intraoperative or postoperative blood transfusion. Under the guidance of preoperative DSCT, satisfactory ischemic areas were achieved by sequentially clamping two (81.8%) or three (18.2%) target SRAs with mean clamping time of 18.8 (range: 15.0–27.0) min for single lesion, and the mean of total clamping time for single case was 37.5 (range: 32.0–52.0) min. (Table 1).

**Table 1:** Operative outcomes

Variables	n (%) or Mean (range)
Cases with successfully sequential SRA clamping, *n*	11 (91.7)
Operative time*, min*	91.7 (72.0–155.0)
Total clamping time for single case*, min*	37.5 (32.0–52.0)
Clamping time for single lesion, *min*	18.8 (15.0–27.0)
Clamped SRAs for single case*, n*	
2	9 (81.8)
3	2 (18.2)
Distribution of target SRAs, *n*	
Anterior	7 (63.6)
Combined (anterior and posterior)	4 (36.4)
Total renorrhaphy time for single case*, min*	20.8 (16.0–27.0)
Estimated blood loss*, ml*	150.0 (50.0–350.0)
Postoperative drainage volume*, ml*	125.0 (70.0–350.0)
Postoperative hospital stay*, day*	4.9 (4.0–7.0)
Complications, *n*	
Functional prolonged ileus (grade 1)	2 (18.2)
Low-grade fever (grade 1)	1 (9.1)
Gross hematuria (grade 2)	1 (9.1)
Pathological findings, *n*	
Clear cell carcinoma	7 (63.6)
Papillary cell carcinoma	1 (9.1)
AML	3 (27.3)
Postoperative Scr^a^*,* μmol*/l*	103.6 (77.9–124.2)
Postoperative eGFR^a^*, ml/minute/1.73m*^*2*^	71.2 (52.7–106.4)

Abbreviation: *SRA* segmental renal artery, *AML* angiomyolipoma, *Scr* Serum creatinine, *eGFR* estimated glomerular filtration rate

^a^Values were tested or calculated on the postoperative 30th day

The mean volume of postoperative drainage was 125.0 (range: 70.0–350.0) ml, and the mean hospital stay after operation was 4.9 (4.0–7.0) days. Only the complications of grade 1–2 were found in the cohort without any one of grade 3–5, and one case had gross hematuria and recovered with only drug treatment. Pathological diagnoses were consistent with preoperative imaging diagnoses in all case and no positive surgical margin was discovered (Table 1). Only one tumor had invasion of the perinephric fat (stage pT3a), and all other tumors were stage pT1a- pT1b in eight cases with RCCs.

The mean follow-up time was 5.4 (range: 2.0–8.0) months and no further embolization or surgical procedure was required. All patients had a normal value of Scr (range: 77.9–124.2 μmol/l) on the postoperative 30th day, and the mean postoperative eGFR was 71.2 ml/minute/1.73m^2^ that was only an insignificant reduction (9.3%) compared with the preoperative baseline (Table 1). No local recurrence or metastasis was found during the follow-up.

## Discussion

The goal of managing MIRTs is to prevent renal tumors dissemination and maximize nephron sparing and postoperative function outcomes. With the development of robot-assisted platform and minimally invasive surgery techniques, the one-time resection of MIRTs with nephron sparing is becoming possible. Warm ischemia (WI) injury plays a principal role that influences the postoperative function outcomes in NSS, and the time of WI should be less than 20 min [15, 22]. Currently, many novel techniques have emerged to decrease WI injury, which are laudable endeavors [11–15, 23–26]. One of main methods is to convert total parenchymal ischemia to local ischemia [11–15]. In 2010, our center first successfully applied LPN with SRA clamping for the treatment of RCCs that has been proved safe and feasible [15]. The technique can minimize the scope of intraoperative WI injury and provide better postoperative function outcomes [12]. Logically, WI time during partial nephrectomy (PN) for MIRTs would be the accumulation of multiple single-lesion processing time if the MRA clamping is applied. Thus the WI time of spared nephrons is less likely to be controlled within 20 min, which will inevitably damage the postoperative function outcomes.

Fortunately, we found a considerable proportion of MIRT patients had at least two SRAs feeding different lesions by preoperative high-quality 3D-DSCT angiography. According to the anatomic characteristic between SRAs and multiple lesions, we originally applied the sequential clamping technique of target SRAs in RPN to avoid the WI time of any spared nephrons more than 20 min. In our cohort, although the mean of total clamping time for single case was 37.5 min, the mean clamping time for single lesion is only 18.8 min that successfully prevents spared nephrons from severe WI injury. All the patients had a normal value of Scr on the postoperative 30th day and the mean postoperative eGFR only had an insignificant reduction compared with the preoperative baseline. (Table 1).

Relying on the advantages of precision, flexibility and stability, the robotic-assisted laparoscopic system facilitates surgeons to perform many complex procedures, like precise hilar microdissection and one-time resection/renorrhaphy for multiple renal lesions. Compared with our previous report on LPN procedure in 2017 [15], RPN with sequential SRA clamping shows more advantages. In despite of the larger tumor size (mean 2.7 vs. 2.5 cm) and higher R.E.N.A.L score (mean 5.7 vs. 4.4) in RPN cohort than in LPN, RPN cohort manifests the better operative outcomes, such as less blood loss (mean 150 vs. 190 ml), shorter operation time (mean 91.7 vs 125 min) and clamping time for single lesion (mean 18.8 vs. 23 min). Therefore, it is foreseeable that the technique of sequential SRA clamping should be more powerful in RPN than in LPN for treating MIRTs.

To our knowledge, this is the initial report of the sequential SRA clamping technique in the procedure of RPN. There have been several reports that described the performance of RPN for MIRTs with MRA clamping or no clamping [24, 27–29]. However, MRA clamping of more than 20 min may cause severe renal ischemia/ reperfusion injury and function damage [27, 28]. At the other extreme, no clamping of renal hilum may lead to more blood loss, greater risk of open procedure or total nephrectomy, and longer surgery time than hilar control [24, 29]. Compared to the above two techniques, SRA clamping only blocks the blood flow of the selected renal segment located by lesions and preserves the supply to other segments. Furtherly, sequential clamping of multiple SRAs can effectively limit the WI time of each involved segment within the recommended 20 min for the one-time resection of MIRTs. Thus this technique minimizes WI injury to the whole kidney by the alternate conversion of ischemic regions, as is evidenced by the insignificant decrease of the postoperative eGFR in our cohort. Moreover, this technique provides better visualization than no clamping, and may help resect less normal parenchyma and avoid positive margins. In our cohort, the mean EBL was 150.0 ml without any intraoperative or postoperative blood transfusion. Only the complications of grade 1–2 were found and no positive surgical margin was discovered.

In this study, we describe our initial experience with sequential SRA clamping in RPN for MIRT treatment. A limitation of this technique is the target SRAs should be isolated precisely. However, SRAs could be dissected in only half of cases, which indicates this technique may not be feasible in certain instances such as cases with dense perirenal fat or short segmental arteries [30]. Moreover, during the NSS study, we did find some MIRT cases unsuitable for sequential SRA clamping before surgery or in operation. For instance, preoperative DSCT revealed only unique SRA feeding multiple lesions or clamping multiple SRAs could not obtain satisfactory ischemic area. Now we have been collecting such cases to compare with those successfully undergoing sequential SRA clamping, and the results from the comparison will be reported in our next article.

Although the advantages of this technique were discovered in this report, some shortcomings should be taken into consideration. First of all, the report only was a retrospective description of single center with a small sample size of twelve cases, for which some complication rates could not be evaluated. Second, we didn’t compare the technique with other approaches to PN, so some more reliable and robust results couldn’t be achieved. Our report only indicates the technique may facilitate a minimally invasive approach to NSS in patients with MIRTs. Third, the mean follow-up time was only 5.4 months. The long-term recurrence rate couldn’t be evaluated due to the lack of a long-term follow-up. Thus large-scale multicenter clinical studies are still needed to further prove the advantages of RPN with sequential SRA clamping in MIRT treatment.

## Conclusion

This novel nephron-sparing technique, RPN with sequential SRA clamping, represents a good alternative for selected patients with MIRTs. With the guidance of DSCT and skilled robotic experience, this technique can facilitate the challenges of minimally invasive approaches to NSS for MIRT patients, who expect a long-term survival while maintaining adequate renal function. The technique is feasible, can be performed in security and maximize renal function preservation. Hence, more cases, longer follow-up, and comparisons with other approaches to PN are needed to confirm these initial outcomes.

## Additional files

Additional file 1: **Figure S1.** The pictures of preoperative computed tomography (CT) scanning and three-dimensional reconstruction (C) show a 2.4*2.7 cm tumor (A) and a 2.1*2.3 cm tumor (B) co-locating in the right kidney of a 57-year-old woman. (TIF 6027 kb)

Additional file 2: **Figure S2.** The schematic drawing (A) and operative photograph (B) of patient positioning and port placement for right robot-assisted partial nephrectomy. IC = iliac crest; CM = costal margin; Ro = robotic port; As = assistant port; Cam = camera port. (TIF 4531 kb)

Additional file 4: **Table S1.** Demographics and imaging features of tumors. (DOCX 20 kb)
