# Supplementary figures and images for: Robotic-assisted partial nephrectomy with sequential clamping of segmental renal arteries for multiple ipsilateral renal tumors: initial outcomes
Source: BMC Urol. 2019 May 3;19:31. doi: 10.1186/s12894-019-0451-y (PMC6500028; doi:10.1186/s12894-019-0451-y)

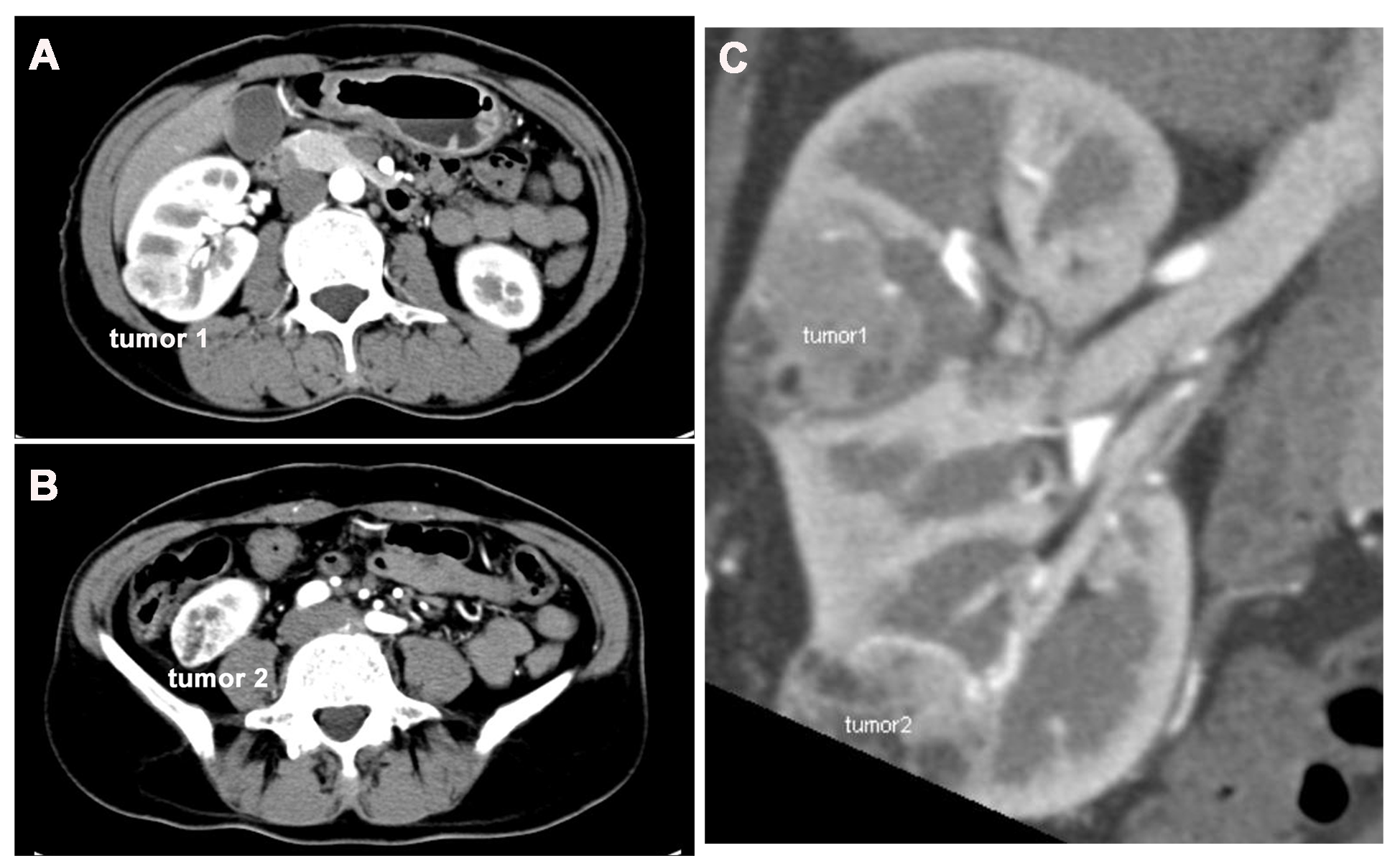

Supplement: Supplementary file 2 — Figure S1. The pictures of preoperative computed tomography (CT) scanning and three-dimensional reconstruction (C) show a 2.4*2.7 cm tumor (A) and a 2.1*2.3 cm tumor (B) co-locating in the right kidney of a 57-year-old woman. (TIF 6027 kb) [file 12894_2019_451_MOESM1_ESM.tif]

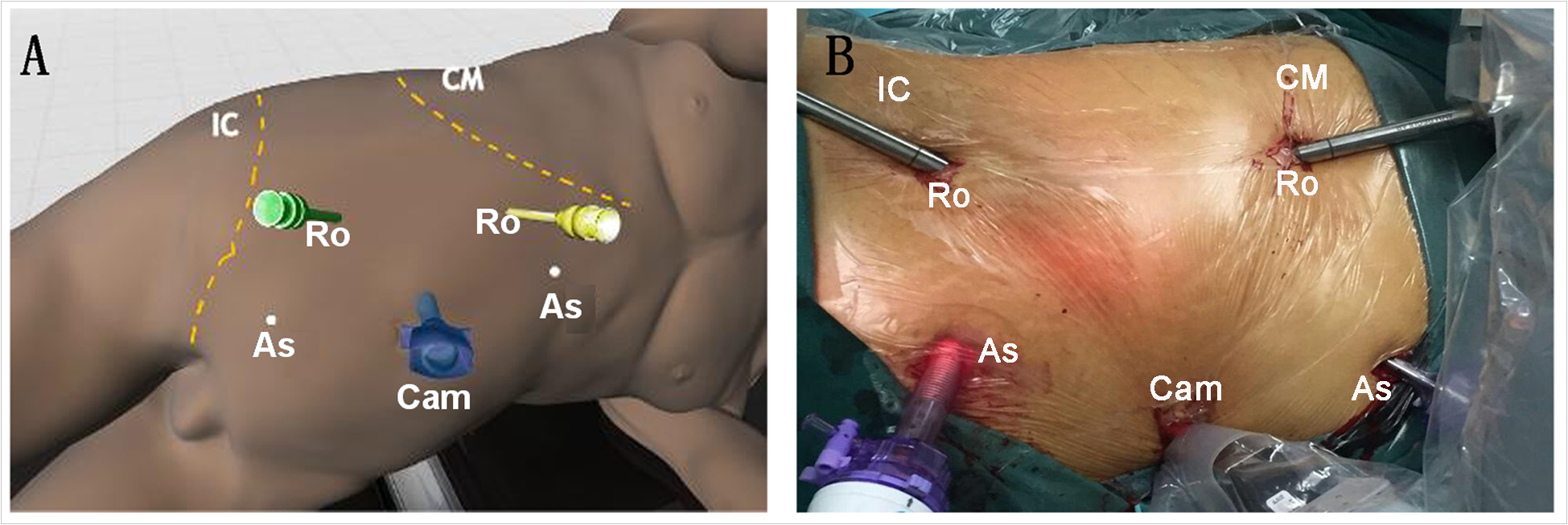

Supplement: Supplementary file 3 — Figure S2. The schematic drawing (A) and operative photograph (B) of patient positioning and port placement for right robot-assisted partial nephrectomy. IC = iliac crest; CM = costal margin; Ro = robotic port; As = assistant port; Cam = camera port. (TIF 4531 kb) [file 12894_2019_451_MOESM2_ESM.tif]
