# Supplementary material for: Robotic-assisted partial nephrectomy with sequential clamping of segmental renal arteries for multiple ipsilateral renal tumors: initial outcomes
Source: BMC Urol. 2019 May 3;19:31. doi: 10.1186/s12894-019-0451-y (PMC6500028; doi:10.1186/s12894-019-0451-y)
Supplement: Supplementary file 4 — Table S1. Demographics and imaging features of tumors. (DOCX 20 kb) [file 12894_2019_451_MOESM4_ESM.docx]

**Supplementary Table 1.** Demographics and imaging features of tumors

| **Variables** | **n (%) or Mean (range)** |
| --- | --- |
| Total cases, *n* | 12 |
| Gender (Male/Female) | 5 (41.7)/ 7 (58.3) |
| Mean age, *years* | 62.7 (42‑77) |
| BMI, *kg/m^2^* | 22.9 (19.2‑ 26.4) |
| ASA score | 1.6 (1-3) |
| Preoperative Scr, *μmol/l* | 92.8 (74.1-122.3) |
| Preoperative eGFR*, ml/minute/1.73m^2^* | 78.5 (54.8-113.0) |
| Total tumor lesions, *n* | 26 |
| Cases with 2 lesions, *n* | 10 (83.3) |
| Cases with 3 lesions, *n* | 2 (16.7) |
| Preoperative radiologic diagnosis*, n* |  |
| RCCs | 9 (75.0) |
| AMLs | 3 (25.0) |
| Single tumor size, *cm* | 2.7 (1.4 - 5.8) |
| Dominant tumor size for single case^1^, *cm* | 3.3 (2.4 - 5.8) |
| Dominant R.E.N.A.L score^1^ | 5.7 (4-10) |
| Locations of lesions for single case, *n* |  |
| Anterior and upper/inferior polar | 6 (50.0) |
| Posterior and upper/inferior polar | 2 (16.7) |
| Upper and inferior polars | 4 (33.3) |
| Growth pattern of lesions for single case, *n* |  |
| All exophytic | 5 (41.7) |
| All mesophytic | 2 (16.7) |
| Exophytic and mesophytic | 4 (33.3) |
| Exophytic and endophytic | 1 (8.3) |
| SRAs for single lesion, *n* |  |
| Lesions with 1 feeding SRA | 22 (84.6) |
| Lesions with 2 feeding SRAs | 4 (15.4) |

Abbreviation: BMI= body mass index; ASA=Anesthesiologists Society of America; Scr=Serum creatinine; eGFR=estimated glomerular filtration rate; AML= angiomyolipoma; RCC= renal-cell carcinoma; SRAs= segmental renal arteries.

^1^ This is the maximum one for each patient.
